# Effect of F18 Bioactive Glass-Treated Gutta-Percha on Interaction with Endodontic Sealers

**DOI:** 10.1590/0103-644020256767

**Published:** 2026-02-02

**Authors:** David Hernández Maldonado, José Leandro de Abreu Jampani, Airton Oliveira Santos-Júnior, Marina Trevelin Souza, Edgar Dutra Zanotto, Juliane Maria Guerreiro-Tanomaru, Mário Tanomaru-Filho

**Affiliations:** 1 Department of Restorative Dentistry, São Paulo State University(UNESP), School of Dentistry, Araraquara, São Paulo, Brazil; 2 Department of Materials Engineering, Vitreous Materials Laboratory (LaMaV), Federal University of São Carlos (UFSCar), São Carlos, São Paulo, Brazil

**Keywords:** adhesion, bioactive glass, biomineralization, gutta-percha, root canal filling materials

## Abstract

This study evaluated the effect of F18 bioactive glass applied to gutta-percha by dip-coating or incorporation on surface bioactivity and bond strength to Bio-C Sealer (BCS) and AH Plus (AHP). Gutta-percha discs (n = 3 per group) were dip-coated with F18 bioactive glass at 2.5%, 5%, and 10% by weight and analyzed by SEM and EDS before and after 28 days of immersion in PBS. Uncoated gutta-percha served as the control for analyses. For the tensile bond strength test, twelve specimens per group (n = 12) were prepared using unmodified gutta-percha, 5% F18 dip-coated gutta-percha, or 2% F18-incorporated gutta-percha, each tested with BCS and AHP. Unmodified gutta-percha with each sealer served as the control. Bond strength was measured using a universal testing machine and analyzed by two-way ANOVA and Tukey's test (α = 0.05). After PBS immersion, the 5% and 10% dip-coated groups exhibited more pronounced F18 deposition and the formation of continuous bioactive surface layers, whereas the control group showed no such features. Regarding bond strength, the 5% F18 dip-coated gutta-percha showed higher values with BCS compared to the control (p < 0.05), and the 2% F18-incorporated gutta-percha also increased bond strength to BCS versus the control (p < 0.05). For AHP, 5% dip-coating reduced bond strength compared with the control (p < 0.05), whereas 2% incorporation did not differ (p > 0.05). F18 bioactive glass promoted surface mineralization of gutta-percha and improved its bond strength to Bio-C Sealer, whether applied by dip-coating or incorporation.

## Resumo

Este estudo avaliou o efeito do biovidro F18 aplicado à guta-percha por meio de dip-coating ou incorporação sobre a bioatividade superficial e a resistência de união aos cimentos Bio-C Sealer (BCS) e AH Plus (AHP). Discos de guta-percha (n = 3 por grupo) foram revestidos com biovidro F18 nas concentrações de 2,5%, 5% e 10% em peso e analisados por MEV e EDS antes e após 28 dias de imersão em PBS. Guta-percha não tratada foi utilizada como controle para as análises. Para o teste de resistência de união por tração, doze espécimes por grupo (n = 12) foram preparados utilizando guta-percha não modificada, guta-percha revestida com 5% de F18 ou guta-percha incorporada com 2% de F18, sendo cada tipo testado com BCS e AHP. Guta-percha não modificada com cada cimento serviu como controle. A resistência de união foi mensurada em máquina universal de ensaios e analisada por ANOVA dois fatores e teste de Tukey (α = 0,05). Após imersão em PBS, os grupos revestidos com 5% e 10% apresentaram deposição mais pronunciada de F18 e formação de camadas bioativas contínuas, enquanto o grupo controle não exibiu tais características. Quanto à resistência de união, a guta-percha revestida com 5% de F18 apresentou valores superiores com BCS em comparação ao controle (p < 0,05), e a guta-percha incorporada com 2% de F18 também aumentou a resistência de união ao BCS em relação ao controle (p < 0,05). Para o AHP, o revestimento com 5% reduziu a resistência de união em comparação ao controle (p < 0,05), enquanto a incorporação de 2% não apresentou diferença (p > 0,05). O biovidro F18 promoveu mineralização superficial da guta-percha e melhorou sua resistência de união ao Bio-C Sealer, tanto quando aplicado por dip-coating quanto por incorporação.

**Figure:**
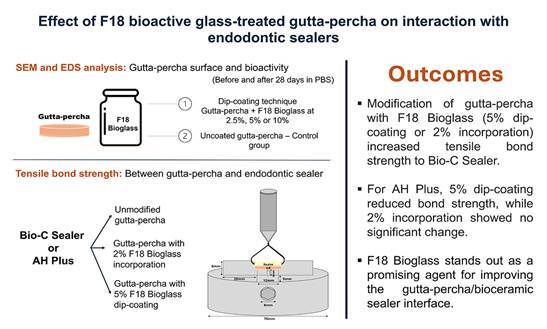

## Introduction

Three-dimensional obturation of root canals is essential for the success of endodontic treatment, preventing bacterial microleakage ^1^. Gutta-percha is the solid material most widely used for this purpose. However, it does not establish adhesive interaction with endodontic sealers, which may compromise apical sealing ^2^ ^,^ ^3^. To overcome this limitation, bioactive compounds have been incorporated into gutta-percha to confer biological potential and improve its mechanical properties ^4^ ^,^ ^5^ ^,^ ^6^. Among these approaches, gutta-percha modified with niobium phosphate bioglass has demonstrated bioactivity, evidenced by the formation of a hydroxyapatite layer on its surface after immersion in simulated body fluid ^6^. This finding suggests that the incorporation of bioglass into gutta-percha may represent a promising alternative by conferring bioactive characteristics to the material ^4^ ^,^ ^5^ ^,^ ^6^. Nevertheless, interfacial failures between gutta-percha and endodontic sealers are still reported in the literature, which may compromise the prognosis of endodontic treatment ^7^ ^,^ ^8^ ^,^ ^9^ ^,^ ^10^ ^,^ ^11^.

Bioactive materials have been extensively investigated in Endodontics due to their biocompatibility and ability to induce biomineralization ^12^. F18® Bioglass (Vetra Biomaterials Ltda., Ribeirão Preto, SP, Brazil), developed by the Vitreous Materials Laboratory (LaMaV), Department of Materials Engineering, Federal University of São Carlos (UFSCar, São Carlos, SP, Brazil), is a highly reactive bioactive glass belonging to the silicon dioxide (SiO₂), sodium oxide (Na₂O), potassium oxide (K₂O), magnesium oxide (MgO), calcium oxide (CaO) and phosphorus pentoxide (P₂O₅) system. This composition provides high thermal stability and a wide processing window, preventing crystallization during processing while maintaining bioactivity ^13^ ^,^ ^14^ ^,^ ^15^. Its ionic balance allows the sustained release of calcium (Ca²⁺), sodium (Na⁺), potassium (K⁺), magnesium (Mg²⁺), and phosphate (PO₄³⁻) ions, promoting rapid hydroxycarbonate apatite deposition and adequate biological bonding with dental tissues ^14^ ^,^ ^15^. Previous studies demonstrated the in vitro and in vivo biocompatibility of F18 bioactive glass and its ability to promote complex and soft tissue formation, confirming its high biological performance ^13^ ^,^ ^14^ ^,^ ^15^. Compared with niobium- or 45S5-based bioactive glasses, F18 combines structural stability with sustained ion release, enhancing apatite precipitation and interfacial biological interaction ^13^ ^,^ ^14^ ^,^ ^15^. Moreover, it exhibits antimicrobial activity, with bactericidal and bacteriostatic effects related to pH changes and electrostatic interactions with bacterial cells ^13^. Although its biological properties are well established, the effects of incorporating F18 bioactive glass into gutta-percha on its physicochemical behavior and adhesion to endodontic sealers remain unknown, representing a relevant gap to be addressed.

AH Plus (Dentsply DeTrey, Konstanz, Germany) is an epoxy resin-based endodontic sealer widely used due to its favorable physicochemical properties and is often employed as a reference material in comparative studies ^16^. However, its main limitation is the lack of bioactivity ^17^ ^,^ ^18^. In contrast, bioceramic endodontic sealers exhibit excellent bioactive performance, with the ability to promote tissue repair and induce apatite formation on the dentin surface ^17^ ^,^ ^18^ ^,^ ^19^ ^,^ ^20^. In this context, the modification of gutta-percha with F18 Bioglass emerges as a promising strategy, as it combines the role of the solid filling material with the potential of F18 to stimulate biomineral deposition and enhance the micromechanical anchorage of bioceramic sealers.

Therefore, this study aimed to evaluate whether the modification of gutta-percha with F18 Bioglass, by dip-coating or incorporation, could improve bioactivity and tensile bond strength to Bio-C Sealer and AH Plus. The null hypothesis stated that such modifications would not affect these properties compared with the tested sealers.

## Materials and methods

**Figure 1:**
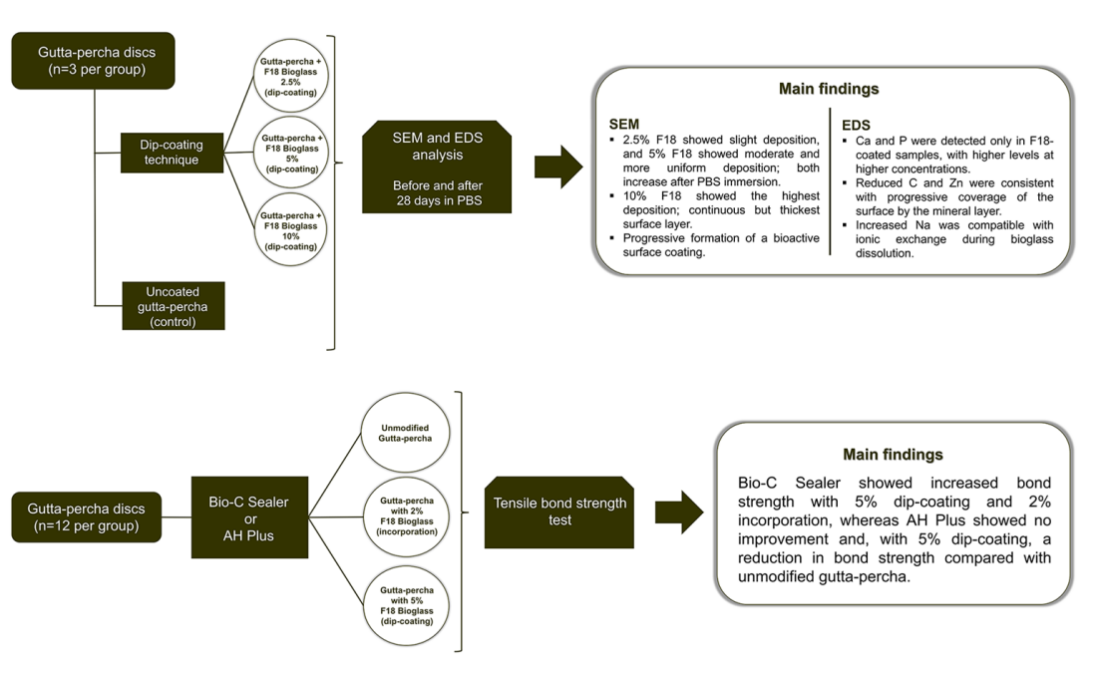
Experimental workflow of the study showing F18 Bioglass treatments, SEM/EDS surface analysis, and tensile bond strength testing.

The materials used in this study, including their manufacturers, compositions, and proportions, are detailed in Table 1. Additionally, Figure 1 presents a workflow summarizing the overall study design.

**Table 1:** Materials, manufacturers, composition, and proportions

Material	Manufacturer	Composition	Proportion
F18® Bioglass	Vetra Biomaterials Ltda., Ribeirão Preto, SP, Brazil; developed by the Vitreous Materials Laboratory (LaMaV), Department of Materials Engineering, Federal University of São Carlos (UFSCar, São Carlos, SP, Brazil)	Silicon dioxide (SiO₂), Sodium oxide (Na₂O), Potassium oxide (K₂O), Magnesium oxide (MgO), Calcium oxide (CaO), Phosphorus pentoxide (P₂O₅) system (oxides in wt%)	Microparticles (D50 ≈ 5 μm) were prepared as suspensions at 2.5%, 5%, and 10% (w/v).
Gutta-percha	Tanariman Ind. Ltda., Manacapuru, AM, Brazil	Gutta-percha 15.2%; resin/wax 2.9%; zinc oxide 81.9%	-
AH Plus	Dentsply DeTrey, Konstanz, Germany	Paste A: Bisphenol‑A epoxy resin; Bisphenol‑F epoxy resin; calcium tungstate; zirconium oxide; silica; iron oxide pigments. Paste B: Dibenzyldiamine; aminoadamantane; tricyclodecane‑diamine; calcium tungstate; zirconium oxide; silica; silicone oil	1 g: 1 g (paste/paste)
Bio‑C Sealer	Angelus, Londrina, PR, Brazil	Calcium silicates; calcium aluminate; calcium oxide; zirconium oxide; iron oxide; silicon dioxide; dispersing agent	Ready‑to‑use

wt % - weight percent; w/v - weight/volume; D₅₀ - median particle diameter.

### Sample Size Determination

The sample size calculation was performed for the tensile bond strength test, based on the study by Al-Haddad et al. (2018) ^4^. An effect size of 0.50, a statistical power of 95%, and a significance level of 5% were adopted. The analysis was conducted using G*Power software version 3.1.7 for Windows (Heinrich-Heine-Universität Düsseldorf, Germany). According to these parameters, each experimental group in the tensile bond strength test included 12 specimens (n = 12). For the scanning electron microscopy (SEM) and energy-dispersive X-ray spectroscopy (EDS) descriptive surface analyses, three specimens per group (n = 3) were used.

### Coating of Gutta-Percha with F18 Bioglass Using the Dip-Coating Technique

Commercial gutta-percha discs (Tanari, Manacapuru, Amazonas, Brazil) with standardized dimensions (11 mm in diameter × 2 mm in thickness) were used. The dip-coating technique was employed to coat the gutta-percha discs with F18 Bioglass at concentrations of 2.5 %, 5 %, and 10 % by weight. The bioglass powder was diluted in a semi-synthetic controlled-release polymeric solution prepared with 0.05 % hydroxypropyl methylcellulose (HPMC) (Federal University of São Carlos, São Carlos, SP, Brazil) using distilled water and isopropyl alcohol in a 1:1 ratio. The solutions were homogenized in a vortex mixer (AP-56, Phoenix Luferco LTDA, Araraquara, SP, Brazil) at 400 rpm for 1 minute. Each gutta-percha disc was identified and immersed twice in the respective solution for 10 seconds each, with an intermediate drying interval of 20 seconds. After coating, the specimens were placed in closed Petri dishes and kept in an incubator at 37 °C for 72 hours to allow solvent evaporation and film stabilization. Finally, the discs were stored in a desiccator until surface and bioactivity analyses.

### Incorporation of F18 Bioglass into Gutta-Percha

Unlike surface dip-coating, the incorporation technique modifies gutta-percha throughout its entire volume, producing a homogeneous composite. Approximately 9 g of gutta-percha (Tanari, Manacapuru, Amazonas, Brazil) were heated in a non-stick container at about 100 °C using a magnetic heater until the material reached a plastic consistency. Subsequently, F18 Bioglass powder (2 % by weight) was gradually added and manually homogenized with a spatula until a uniform mixture was obtained. The softened material was then pressed into silicone molds (11 mm in diameter × 4 mm in thickness) and allowed to cool to room temperature for solidification. The discs were demolded and stored in a desiccator at room temperature until testing. The resulting specimens were used for the tensile bond strength analysis (n = 12 per group).

### Surface Analysis of Gutta-Percha and Bioactivity under SEM

Gutta-percha discs (Tanari) with a diameter of 11 mm and a thickness of 2 mm were prepared. The specimens were divided into four experimental groups: uncoated gutta-percha (control) and gutta-percha coated with F18 Bioglass at concentrations of 2.5%, 5%, and 10% by weight. For partial coating, eighteen discs were immersed halfway into a 0.05% hydroxypropyl methylcellulose solution containing F18 Bioglass. After this process, they were kept in a desiccator for 72 hours. Another eighteen discs were fully coated with F18 Bioglass at the same concentrations and stored in an incubator at 37 °C for 72 hours. Subsequently, all specimens were placed in plastic tubes containing 10 mL of phosphate-buffered saline (PBS) and maintained for 28 days at 37 °C. After this period, they were again stored in a desiccator for 14 days. Before microscopic analysis, the discs were cleaned, dried, and prepared following standard procedures for surface examination. Each specimen was subsequently sputter-coated with gold to allow surface conductivity during scanning electron microscopy (SEM) observation. Three independent discs per group (n = 3) were analyzed using a scanning electron microscope (JEOL JSM-6610LV, Tokyo, Japan). For each disc, four distinct surface areas were examined, totaling 12 analyses per group. The evaluation included specimens without PBS immersion and after 28 days of immersion. Images were qualitatively analyzed to identify F18 Bioglass deposition and surface changes on gutta-percha. Surface classification was performed according to previously established scoring criteria (Table 2).

**Table 2:** Score definitions for the percentage of the gutta-percha surface covered by F18 Bioglass

Score	Description
1	Slight coverage of the gutta‑percha by F18 Bioglass
2	F18 Bioglass covering 25% of the gutta‑percha surface
3	F18 Bioglass covering 50% of the gutta‑percha surface
4	F18 Bioglass covering 75% of the gutta‑percha surface
5	F18 Bioglass covering more than 75% of the gutta‑percha surface

### Bioactivity Analysis by EDS

The same three gutta-percha discs per group (n = 3) used for SEM analysis were also evaluated by EDS. The EDS measurements were performed in the same SEM session, immediately after surface imaging and without removing the specimens from the microscope chamber. For each disc, three representative surface points were selected based on the corresponding SEM micrographs, and semi-quantitative elemental spectra were acquired using the standard spot analysis mode of the equipment (approximately 1-2 µm in diameter). This procedure enabled the identification and semi-quantification of elements associated with bioactive behavior, such as calcium and phosphorus, after the different F18 Bioglass treatments and following 28 days of PBS immersion.

### Tensile Bond Strength Between Endodontic Sealer and Gutta-Percha

The tensile bond strength test was performed using 12 independent specimens per group (n = 12). Automotive resin discs with a diameter of 15 mm and a thickness of 4 mm were fabricated, each containing a central cavity measuring 11 mm in diameter and 1 mm in thickness. After standardization and polishing, the cavities were filled with the endodontic sealers according to the experimental groups.

Based on the SEM results, the 5% concentration of F18 Bioglass was selected exclusively for the dip-coating treatment used in the tensile bond strength test. For this evaluation, three types of gutta-percha discs were prepared: untreated gutta-percha (control), gutta-percha incorporated with 2% F18 Bioglass, and gutta-percha dip-coated with 5% F18 Bioglass. Each type of disc was placed in contact with the respective endodontic sealer (Bio-C Sealer or AH Plus) while the material was still freshly mixed and unset, ensuring intimate and reproducible contact consistent with clinical conditions. This process resulted in six experimental groups: untreated gutta-percha with Bio-C Sealer; 2% F18-incorporated gutta-percha with Bio-C Sealer; 5% F18 dip-coated gutta-percha with Bio-C Sealer; untreated gutta-percha with AH Plus; 2% F18-incorporated gutta-percha with AH Plus; and 5% F18 dip-coated gutta-percha with AH Plus.

**Figure 2:**
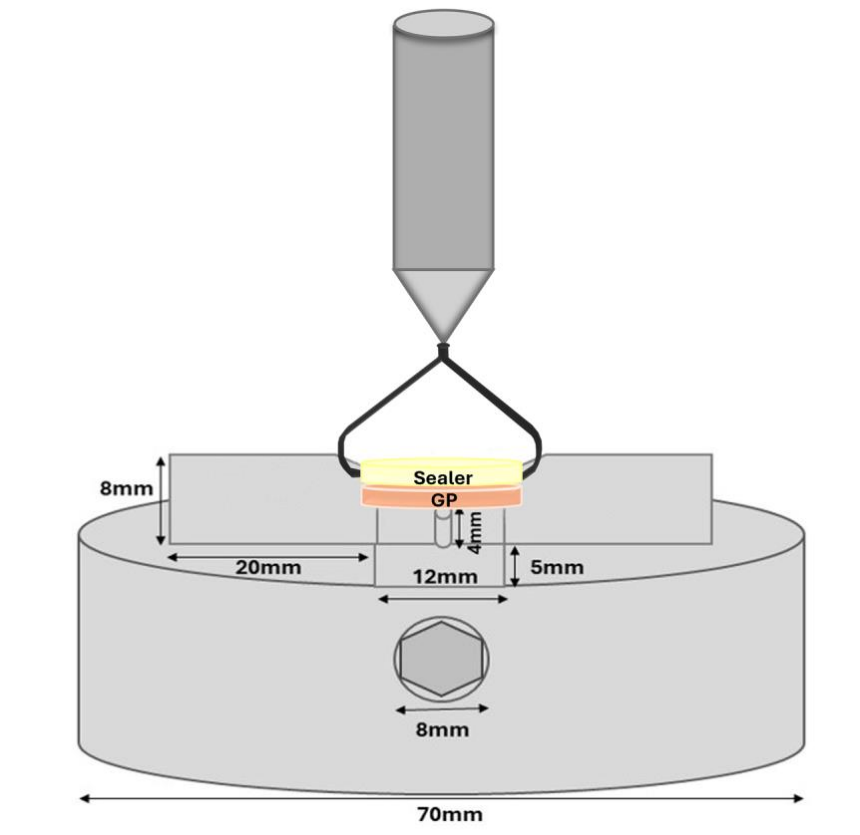
Schematic representation of the custom-designed device developed for tensile bond strength testing.

In the AH Plus groups, the specimens were stored in an incubator at 37 °C under relative humidity for 7 days. In the Bio-C Sealer groups, the specimens were initially placed in containers with gauze moistened with distilled water for 24 hours and subsequently immersed in 2 mL of distilled water at 37 °C for 7 days. After the storage period, each specimen was positioned in a device developed by the authors (Figure 2), specifically designed to fit the universal testing machine (Emic DL 2000, São José dos Pinhais, Brazil). Tensile testing was performed using a 1-kilonewton (kN) load cell at a constant speed of 0.5 mm/min. In this system, the gutta-percha disc was fixed to the lower base of the device, while the sealer disc was attached to the upper portion by a metal loop, allowing controlled application of tensile load (Figure 2). The maximum load at failure (N) was recorded and converted into bond strength (MPa) using the formula ^4^:

**Figure:**
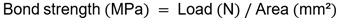

where the bonding area corresponded to the cavity diameter of 11 mm (95.03 mm²).

After tensile testing, the specimens were examined under a stereomicroscope to determine the mode of failure, which was classified as adhesive qualitatively (absence of sealer adhered to the gutta-percha surface), cohesive (presence of sealer remnants on the gutta-percha surface), or mixed (a combination of both patterns).

### Statistical Analysis

The data were analyzed using two-way ANOVA considering the factors sealer type and gutta-percha modification with F18 Bioglass. Tukey’s post hoc test was applied for multiple comparisons at a 5% significance level (p < 0.05). SEM and EDS data were analyzed descriptively.

## Results

### Evaluation of Gutta-Percha Surface and Bioactivity by SEM

**Figure 3:**
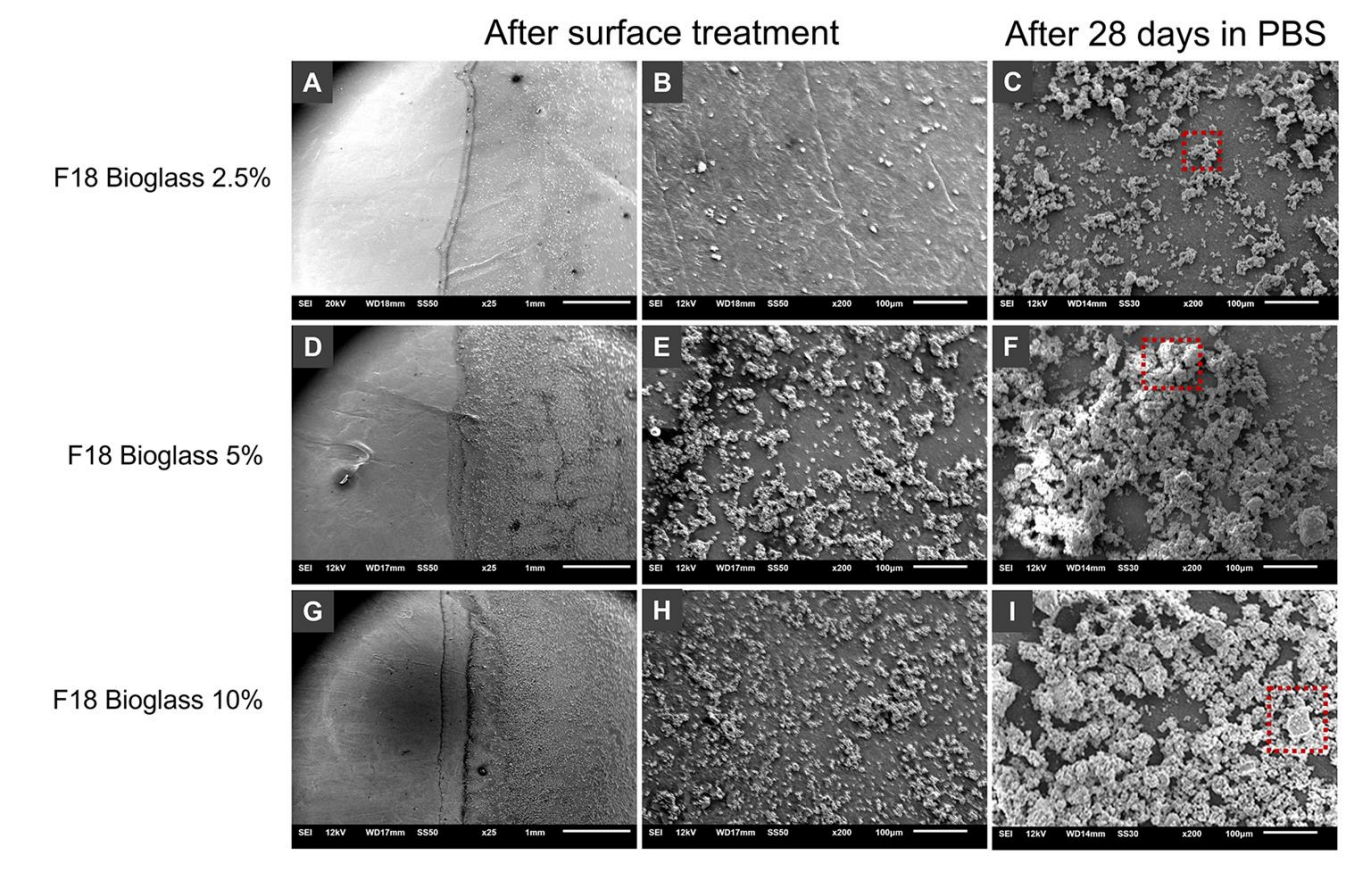
Scanning electron microscopy (SEM) images after surface treatment and after 28 days of immersion in PBS, with magnifications indicated by the scale bars. Gutta-percha dip-coated with 2.5% F18 Bioglass is shown in A-B, and the same group after PBS immersion in C, where mineral deposition is highlighted (red box). Specimens treated with 5% F18 Bioglass appear in D-E, and after 28 days in PBS in F, showing a thicker mineral layer (red box). Gutta-percha coated with 10% F18 Bioglass is presented in G-H, and the corresponding post-immersion surface in I, displaying the highest degree of coverage (red box).

In the specimens coated with 2.5% F18 Bioglass, a slight deposition of the material was observed, corresponding to score 1 **(** Figure 3, A and B). After immersion in PBS for 28 days, deposition increased, covering approximately 25% of the surface, which corresponded to score 2 (Figure 3, C).

In the 5% F18 Bioglass group, the initial coverage reached about 50% of the surface, classified as score 3 (Figure 3, D and E). After PBS immersion, deposition increased, covering between 50% and 75% of the surface, corresponding to score 4 (Figure 3, F).

In the specimens coated with 10% F18 Bioglass, the initial coverage ranged from 50% to 75% of the surface, classified as score 4 (Figure 3, G and H). After immersion, deposition exceeded 75% of the surface, resulting in a score of 5 (Figure 3, I).

### Bioactivity Analysis by EDS

EDS revealed relevant changes in the elemental composition of the gutta-percha surface after coating with F18 Bioglass and immersion in PBS for 28 days. In the control group, calcium and phosphorus were not detected. In contrast, these elements became evident in the treated specimens, with lower values in the 2.5% coating, intermediate values in the 5% coating, and higher values in the 10% coating (Table 3). In addition, the treated groups showed a progressive reduction in zinc content, a decrease in carbon levels, and an increase in sodium concentration compared with the control. Such deposits are consistent with the precipitation of calcium phosphate phases, suggesting the bioactive potential of F18 Bioglass.

**Table 3:** Elemental composition (%) of gutta-percha surfaces after 28 days of immersion in PBS, determined by energy-dispersive X-ray spectroscopy (EDS), under different F18 Bioglass treatments

Groups	C (%)	O (%)	Zn (%)	Na (%)	P (%)	Ca (%)
F18 Bioglass 2.5%	36.5	10.9	23.5	12.9	0.8	0.8
F18 Bioglass 5%	38.1	8.8	20.0	11.2	4.9	9.5
F18 Bioglass 10%	25.0	9.5	10.0	13.1	5.1	10.7
Untreated gutta-percha	44.5	11.3	40.7	3.5	0.0	0.0

PBS: phosphate-buffered saline, C: carbon, O: oxygen, Zn: zinc, Na: sodium, P: phosphorus, Ca: calcium. Values are means of EDS measurements acquired from three areas per specimen (n=3 per group), expressed as percentages.

### Tensile Bond Strength and Failure Mode Analysis

Table 4 shows that dip-coating gutta-percha with 5% F18 Bioglass significantly increased tensile bond strength to Bio-C Sealer compared with the control group (untreated gutta-percha) (p < 0.05). However, for AH Plus, dip-coating with 5% F18 Bioglass significantly reduced bond strength compared with the control (p < 0.05). In addition, untreated gutta-percha showed lower bond strength to Bio-C Sealer than to AH Plus (p < 0.05). No significant differences were observed between the groups dip-coated with 5% F18 Bioglass, regardless of the sealer used (p > 0.05).

The incorporation of 2% F18 Bioglass into gutta-percha significantly increased tensile bond strength to Bio-C Sealer compared with the group without incorporation (p < 0.05). For AH Plus, no significant differences were observed between the incorporated groups and the control (p > 0.05) (Table 4).

**Table 4:** Mean ± standard deviation of tensile bond strength of gutta-percha modified with F18 Bioglass

Test	Coating technique (dip-coating)			
	Bio-C Sealer		AH Plus	
Tensile bond strength (MPa)	GP/BCS	GP + 5% F18	GP/AHP	GP + 5% F18
	0.95 ± 0.31^a^	1.79 ± 0.70^b^	2.25 ± 0.41^c^	1.76 ± 0.61^b^
	Incorporation Technique			
	GP/BCS	GP + 2% F18	GP/AHP	GP + 2% F18
	0.90 ± 0.25^a^	1.62 ± 0.24^b^	2.25 ± 0.41^c^	2.06 ± 0.39^bc^

MPa: megapascal, BCS: Bio-C Sealer, AHP: AH Plus, GP: gutta-percha, GP + 5% F18: gutta-percha coated with 5% F18 Bioglass, GP + 2% F18: gutta-percha incorporated with 2% F18 Bioglass. Different lowercase letters indicate statistically significant differences among groups (p<0.05).

Regarding the failure mode (Table 5), adhesive failures predominated in all groups. Only in the group of gutta-percha dip-coated with 5% F18 Bioglass combined with Bio-C Sealer was a higher incidence of mixed failures observed. No cohesive failures were recorded in any of the groups.

**Table 5:** Percentage (%) of failure modes observed in each experimental group after tensile bond strength testing

Failure mode	GP/BCS	GPI/F18 2%/BCS	GP/F18 5%/BCS	GP/AHP	GPI/F18 2% /AHP	GP/F18 5%/AHP
Adhesive	80	64	45	100	91	72
Cohesive	-	-	-	-	-	-
Mixed	20	36	55	-	9	18

GP: gutta-percha, GPI/F18 2%: gutta-percha incorporated with 2% F18 bioactive glass, GP/F18 5%: gutta-percha dip-coated with 5% F18 bioactive glass, BCS: Bio-C Sealer, AHP: AH Plus.

## Discussion

The present study aimed to evaluate the effect of gutta-percha modification with F18 Bioglass, either by dip-coating or incorporation, on the tensile bond strength to Bio-C Sealer and AH Plus. The results showed that the modification, particularly dip-coating with 5% F18 Bioglass, significantly increased bond strength to Bio-C Sealer. Therefore, the null hypothesis was rejected.

SEM analysis in this investigation showed that coating gutta-percha with F18 Bioglass by the dip-coating technique promoted progressive deposition of bioactive particles. Coverage was slight at 2.5%, more evident at 5% (approximately 50% of the surface), and extensive at 10% (50-75%). After 28 days of immersion in PBS, an intensification of deposition was observed in all groups, reaching more than 75% of the surface at the 10% concentration. These findings indicate that the simulated physiological environment promotes ionic release and the formation of bioactive deposits ^18^ ^,^ ^22^. Moreover, they suggest that the dip-coating technique enables the formation of a continuous bioactive coating on gutta-percha. Similar results were observed on zirconia surfaces treated with silica-zirconia by dip-coating, which showed greater bioactivity and bond strength ^23^. Likewise, in titanium implants, hydroxyapatite coatings associated with chitosan obtained by dip-coating promoted apatite formation, cell adhesion, and antibacterial activity ^24^. Although the 10% F18 Bioglass concentration exhibited the highest mineral deposition in this study, a more detailed inspection showed that this thicker layer produced a more pronounced surface topography. Because the tensile bond strength test requires standardized and reproducible surface contact between the gutta-percha and the sealer, the 5% concentration was selected for mechanical testing, as it generated a more homogeneous and controlled coating while still demonstrating clear bioactive potential.

EDS analysis in this study revealed significant changes in the chemical composition of the gutta-percha surface after coating with F18 Bioglass and immersion in PBS for 28 days. Calcium and phosphorus, absent in the control group, were detected in the treated specimens, confirming the formation of deposits consistent with calcium phosphate phases (Table 3). The progressive increase in these elements, together with the reduction in zinc and carbon, suggests partial substitution of the original matrix by the mineralized coating. The increase in sodium content is consistent with ionic exchanges characteristic of bioglass dissolution in a physiological environment ^25^ ^,^ ^26^. Taken together, these findings explain the greater deposition observed under SEM and support the interpretation that the surface becomes progressively covered by a mineralized layer formed through ion release and subsequent precipitation. Such changes indicate surface bioactivity, although they do not allow confirmation of a direct chemical interaction with the underlying gutta-percha, since EDS provides only localized elemental information. However, as complementary analyses such as X-ray diffraction (XRD) or Fourier-transform infrared spectroscopy (FTIR) were not performed in this study, the identification of these phases should be interpreted as an indirect indication of the material’s bioactivity rather than a definitive confirmation of hydroxyapatite formation.

In this study, dip-coating gutta-percha with 5% F18 Bioglass and incorporating 2% F18 increased the tensile bond strength to Bio-C Sealer. EDS analysis showed an increase in calcium and phosphorus and a reduction in zinc and carbon after PBS immersion. Because the measurements were performed on the superficial deposit formed by the F18 Bioglass treatment, these elemental changes reflect the composition of the mineralized surface layer rather than the underlying gutta-percha substrate. Therefore, the findings indicate surface-level mineral precipitation consistent with calcium phosphate deposition, without allowing inferences regarding chemical modification of the internal gutta-percha matrix. This surface mineral layer is compatible with the ion release profile of bioglass and with the alkaline environment generated by calcium silicate-based sealers, both of which favor apatite formation ^27^ ^,^ ^28^ ^,^ ^29^. The resulting deposit may enhance interfacial retention by promoting chemical interaction and micro-anchorage, which aligns with the predominance of mixed failures observed in the treated groups in this study. Similar results were observed in a study with bioceramic cones associated with Bio-C Sealer, which demonstrated higher bond strength and a more stable interface ^30^.

In contrast, the current results revealed that dip-coating gutta-percha with 5% F18 Bioglass significantly reduced tensile bond strength to AH Plus. In addition, incorporation with 2% did not alter the values compared with the control. This behavior may be related to differences in the adhesion mechanisms of the sealers tested in the present study. AH Plus, as an epoxy resin-based sealer, relies mainly on micromechanical anchorage and infiltration of the resinous matrix into the substrate surface ^31^. The mineralized and hydrophilic coating formed by F18 Bioglass may have hindered this penetration, thereby compromising bonding effectiveness ^32^. The predominance of adhesive failures in all evaluated groups confirms that the gutta-percha/AH Plus interface remains a critical point. These findings reinforce that the interaction between AH Plus and gutta-percha is essentially mechanical, with no additional benefit from bioactive coating ^33^.

The findings of this study reinforce the clinical relevance of the gutta-percha/sealer interface, which remains a decisive factor for the success of endodontic treatment ^5^ ^,^ ^6^ ^,^ ^7^. Modification of gutta-percha with F18 Bioglass demonstrated potential to strengthen this interface, particularly when associated with the bioceramic sealer, enhancing sealing ability and increasing the likelihood of clinical success. However, some methodological limitations must be acknowledged. The incorporation technique required heating gutta-percha above its softening temperature, which may have altered its physical behavior relative to clinical conditions. Furthermore, the EDS analysis provided only localized, point-based elemental information, limiting full-surface chemical characterization. In addition, the in vitro model used does not fully reproduce the biological and mechanical complexity of the clinical environment. These aspects should be considered when interpreting the results. Consequently future studies should confirm the formation of chemical bonds, validate the clinical applicability of these modifications, and explore testing models that better simulate clinical scenarios while assessing the long-term behavior of modified gutta-percha.

## Conclusion

In conclusion, modification of gutta-percha by dip-coating or incorporation with F18 Bioglass increases tensile bond strength to the bioceramic sealer Bio-C Sealer. Thus, F18 Bioglass stands out as a promising bioactive agent to optimize the interface between gutta-percha and bioceramic sealers.

## Data Availability

The data that support the findings of this study are available from the corresponding author upon reasonable request.
